# Supplementary material for: Survey research of patient’s preference on choosing microscopic or endoscopic spine surgery for lumbar discectomy
Source: PLoS One. 2023 Apr 6;18(4):e0283904. doi: 10.1371/journal.pone.0283904 (PMC10079117; doi:10.1371/journal.pone.0283904)
Supplement: S1 Appendix — (DOCX) [file pone.0283904.s001.docx]

**Appendix 1**

**Surgical Treatment Options for Lumbar Disc Herniation**

The intervertebral discs are fibrocartilaginous cushions which lie between two vertebrae in the spinal column. Herniated disc occurs when the inner portion of intervertebral disc protrudes through the tear of the outer ring. This gelatinous inner portion can compress and irritate spinal nerve roots. This is most commonly found in age range from 30-50 years old. Acute low back pain with radiation through buttock and down to leg are the main symptoms. Some patients may experience numbness and weakness of lower extremities. In serious cases, the patients may develop dysfunction of bowel and bladder such as incontinence and loss of sensation around the rectum. These symptoms usually occur suddenly follow using their back to lift heavy objects or twisting and bending while lifting.

In patents, who suffer with pain without severe neurological deficits, treatment usually starts with rest, medication, physical therapy or epidural steroid injection. If the patients do not experience relief from those conservative treatments for 4-6 weeks, surgical treatments may be considered. For a few relatively rare conditions such as dysfunction of bowel and bladder or progressive severe neurological deficit, prompt surgical treatment should be performed.

There are several surgical treatment methods for lumbar disc herniation. In this research we focus on the microscopic discectomy and endoscopic discectomy. When compared to conventional open discectomy which needs long incision and bilateral paraspinal muscles dissection, microscopic discectomy and endoscopic discectomy are minimal invasive techniques.

Microscopic discectomy (Fig 1.1) has become the standard treatment of lumbar disc herniation. The operation is performed through a short incision (3-5 cm.). With the greater magnification of the operative field, although the microscopic discectomy is an open surgery, it is done with less stripping of paraspinal muscles than conventional open discectomy. The results of surgery are usually promising. This operation is performed under a general anesthesia.

Full endoscopic discectomy (Fig 1.2) is the operation which is performed through the 8 mm working cannula. This cannula (cylindrical tube) has a small camera at the end of the tube which transfers the signal of pictures to the monitor. Doctor can remove the disc material through this cannula under visualization on the monitor. No muscle stripping is needed and less scarring with minimal blood loss.


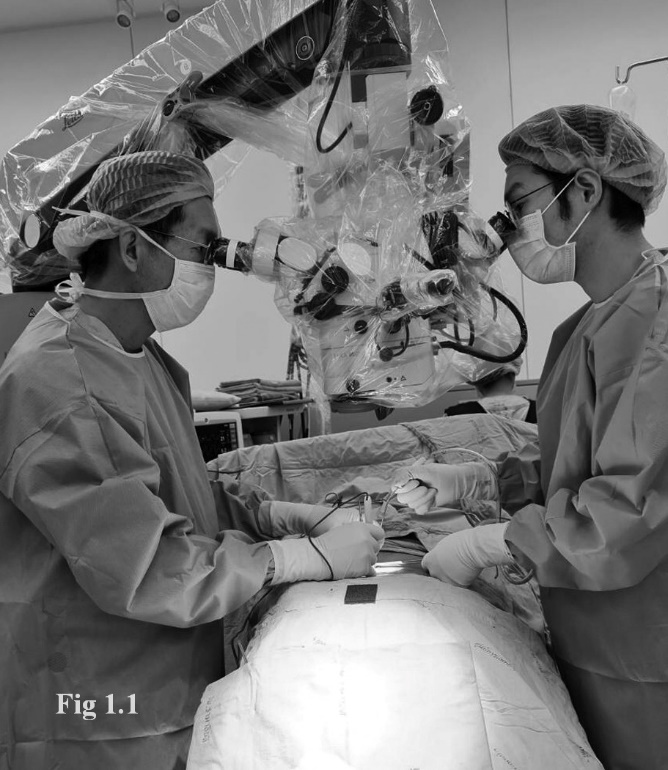


**Fig 1.1** Surgeons use operating microscope to perform microscopic discectomy.


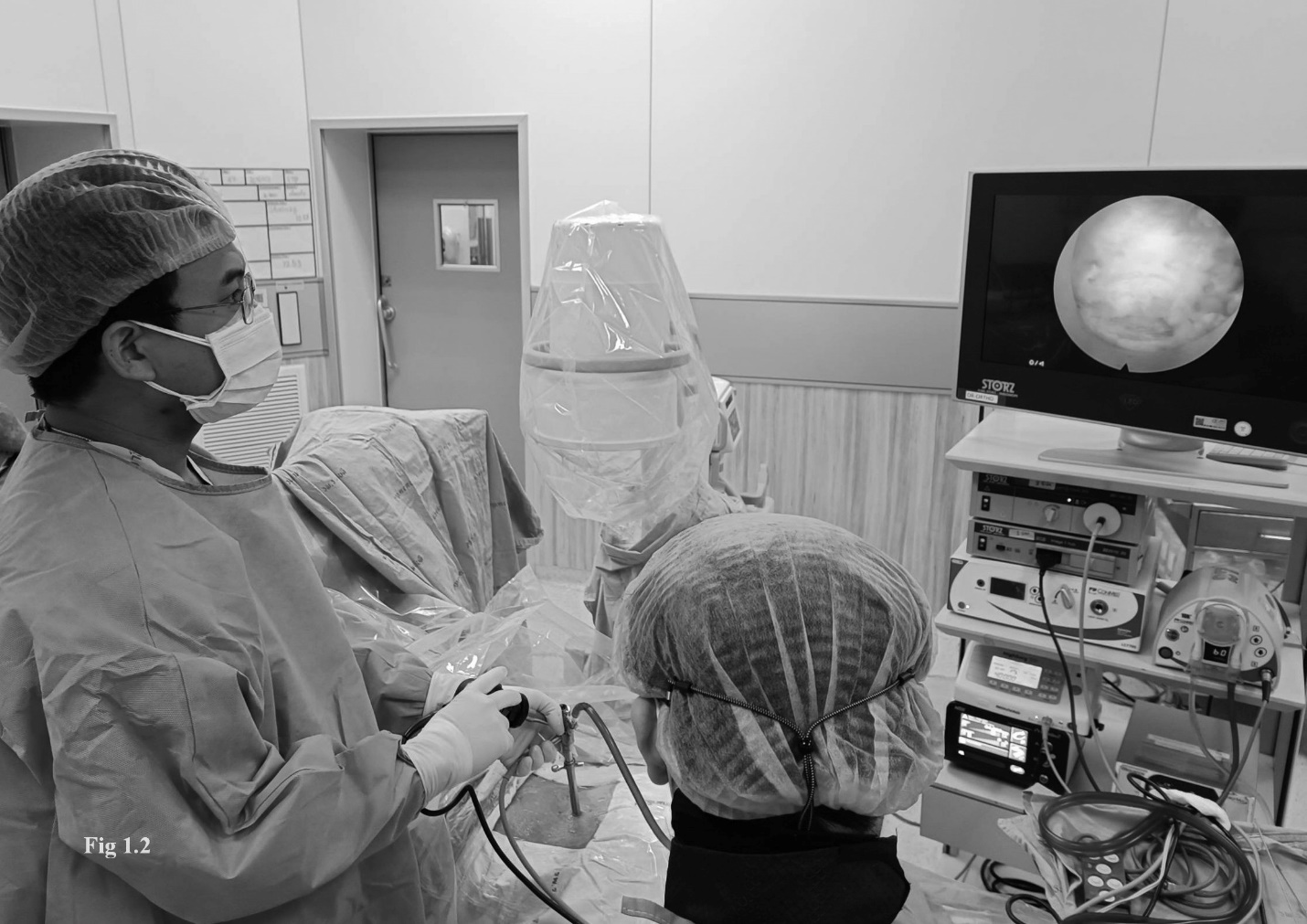


**Fig 1.2** Surgeons use the small hollow cylindrical tube with camera to perform endoscopic discectomy.

Surgical complication rate compared between microscopic discectomy and endoscopic discectomy are inconclusive due to low quality evidence and high risk of bias studies. However, both types of surgery have reported the same types of complications such as dura tear, reherniation, and reoperation. Full endoscopic discectomy found less blood loss (estimated reduction 45 ml), shorter operating time (estimated shorter 12-21 min) and shorter length of stay (estimated shorter 1 day) than microscopic discectomy (Table 1).

Both microscopic discectomy and full endoscopic discectomy are minimal invasive spine surgeries. There is no clear evidence for how to select the procedures. In additional to the clinical outcomes, training and surgeon experience, hospital and equipment are the factors to choose the right operative techniques. Microscopic discectomy needs operating microscope and basic surgical instruments while endoscopic discectomy needs endoscope with long instruments which can be inserted through the hollow cannula and needs special electrocautery for hemostasis. Therefore, cost of endoscopic discectomy is more expensive than microscopic discectomy. Endoscopic discectomy is performed under very small incision with no paraspinal muscle stripping, although the visualization of the operating field is less than microscopic discectomy. To perform endoscopic discectomy, surgeon needs skill to work through the cannula while watching the screen which is more unfamiliar when compared to microscopic discectomy. Therefore, the learning curve of endoscopic discectomy is longer than microscopic discectomy.

In conclusion both techniques provided the similar clinical outcomes, complication rate, reherniation and reoperation. Endoscopic discectomy may need more equipment, more skill and more learning curve. The operative time and length of stay of endoscopic discectomy are significantly less than microscopic discectomy (Table 1).

**Table 1.** Comparing between microscopic discectomy and endoscopic discectomy

| **Microscopic discectomy** |  | **Endoscopic discectomy** |
| --- | --- | --- |
| 3-5 centimeters | **Wound size** | 8 millimeters |
| General anesthesia | **Anesthetic methods** | Local or general anesthesia |
| 43-65 minutes | **Operative time** | 22-57 minutes |
| 44-45 ML | **Blood loss** | 0-33 ML |
| 94% | **Outcomes of treatment (Good to Excellent)** | 98% |
| 0%-2.33% | **Complications** | 0%-4.9% |
| 5.7%-5.94% | **Revision rate** | 6.6%-7.92% |
| 1000 US dollars | **Cost** | 2000 US dollars |
| 2-3 days | **Length of stay** | 0.7-1 day |

From the information which we provide to you, please answer the following question.
