# Supplementary material for: Survey research of patient’s preference on choosing microscopic or endoscopic spine surgery for lumbar discectomy
Source: PLoS One. 2023 Apr 6;18(4):e0283904. doi: 10.1371/journal.pone.0283904 (PMC10079117; doi:10.1371/journal.pone.0283904)
Supplement: S2 Appendix — (DOCX) [file pone.0283904.s002.docx]

**Appendix 2**

**Survey Questionnaire**

**Background**

**Age**  : years

**Sex**  : Female Male

**Status**  : Single Marriage Divorce/Separate

**Address** :

**Education** : High school Bachelor Higher than Bachelor

**Employer type**: Self-employed Company employee Government employee Other

**Gross individual income** :

**Previous Treatment**

Have you ever undergone lumbar discectomy surgery?

- - Yes, microscopic assisted surgery
  - Yes, endoscopic assisted surgery
  - No

**Patient’s Perspective on Surgical Treatment Options**

1. From the summary information sheet, if you have to undergo lumbar discectomy, what kind of surgical treatment do you want to choose?
   - Microscopic assisted surgery
   - Endoscopic assisted surgery
2. In your opinion, from the scale of 1 (least important) to 10 (most important), how much of these factors are important for your surgical treatment choice?

|  | 1 | 2 | 3 | 4 | 5 | 6 | 7 | 8 | 9 | 10 |
| --- | --- | --- | --- | --- | --- | --- | --- | --- | --- | --- |
| Wound size |  |  |  |  |  |  |  |  |  |  |
| Anesthetic methods |  |  |  |  |  |  |  |  |  |  |
| Operative time |  |  |  |  |  |  |  |  |  |  |
| Blood loss |  |  |  |  |  |  |  |  |  |  |
| Outcomes of treatment |  |  |  |  |  |  |  |  |  |  |
| Complications |  |  |  |  |  |  |  |  |  |  |
| Revision rate |  |  |  |  |  |  |  |  |  |  |
| Cost |  |  |  |  |  |  |  |  |  |  |
| Length of stay |  |  |  |  |  |  |  |  |  |  |

1. Have you ever received information about surgical treatment methods for lumbar disc herniation?
   - No  Yes

1. Based on the presented summary information sheet, did this article help in decision making to select your surgical treatment method?
   - Yes, it did  Partially yes  No, it did not **Have you ever undergone lumbar spine surgery, if “Yes” please answer questions nos. 5-8, if “No” please go to question no. 9**

1. Have you ever received the information regarding endoscopic assisted lumbar spine surgery or microscopic assisted lumbar spine surgery before surgery?
   - Yes  No

1. Did you have a chance to select the surgical treatment methods by yourself?
   - Yes  No

1. If you have chance to selected surgical treatment methods again, would you select the same operation which you underwent?
   - Yes  No

1. Would you recommend the same operation which you underwent to other people who need operative treatment?

- - Yes  No

1. How strongly do you feel about your answer? (1 = not strongly 10 = very strongly)

| 1 | 2 | 3 | 4 | 5 | 6 | 7 | 8 | 9 | 10 |
| --- | --- | --- | --- | --- | --- | --- | --- | --- | --- |
|  |  |  |  |  |  |  |  |  |  |
