# Supplementary material for: Survey research of patient’s preference on choosing microscopic or endoscopic spine surgery for lumbar discectomy
Source: PLoS One. 2023 Apr 6;18(4):e0283904. doi: 10.1371/journal.pone.0283904 (PMC10079117; doi:10.1371/journal.pone.0283904)
Supplement: Attachment — Submitted filename: Comments.docx [file pone.0283904.s003.docx]

Thank you for giving me the chance to review the manuscript entitled “Survey research of patient’s preference on choosing microscopic or endoscopic spine surgery for lumbar discectomy”. This is a very interesting and useful study. The experiment was well designed. And the manuscript was well written. It could promote doctors getting a clearer idea of patients’ preferences for surgical approaches. Hence, I recommend accepting the manuscript after a minor revision.

1.	As a spine surgeon, I think some patients are not suitable for ELD, whether such patients excluded from this study?

2.	I think the author could include patients who underwent surgery for one year or more years in future studies. It can help to understand how the long-term outcomes of the two surgical methods influence patients' preferences.

3.	The conclusion is too long, which should be shortened.
