# Supplementary material for: Survey research of patient’s preference on choosing microscopic or endoscopic spine surgery for lumbar discectomy
Source: PLoS One. 2023 Apr 6;18(4):e0283904. doi: 10.1371/journal.pone.0283904 (PMC10079117; doi:10.1371/journal.pone.0283904)
Supplement: Attachment — Submitted filename: Response to Reviewers.docx [file pone.0283904.s004.docx]

Dear	Thamer Hamdan, PhD

Academic Editor

PLOS ONE

We would like to thanks the editor and both reviewers for the comments to our manuscript PONE-D-22-08950 (Survey research of patient’s preference on choosing microscopic or endoscopic spine surgery for lumbar discectomy). We did revise our manuscript following the comments as below. All of the changes were placed in the new upload manuscript. The revised manuscript was submitted in both “Revised Manuscript with Track Changes” and “Manuscript”.

Due to we have Appendix 1 and Appendix 2 with contain information sheet for the patients and the questionnaire. We did not put both Appendices in manuscript because they make manuscript long and difficult to read. If there are links from the manuscript to access Appendix1 and 2 when click on the words, it would be better.

Then we submitted Appendices in “Supporting information” named as S1Appendix and S2 Appendix. Inside S1 Appendix, there are 2 figures and 1 table. To prevent confusion with figures in manuscript, we labeled them as **Fig1.1 in S1Appendix and Fig 1.2 in S1Appendix** respectively.

We added the full name of the ethic committee and also added “All participants provided written informed consent before answering the questionnaires” in Materials and Methods section.

Thank you very much for the consideration.

Sincerely yours,

Chaiwat Kraiwattanapong, MD.

Response to reviewers as follow:

**Reviewer #1:**

**Comment 1: As a spine surgeon, I think some patients are not suitable for ELD, whether such patients excluded from this study?**

**Reply:** In our institute we definitely have exclusion criteria for endoscopic discectomy such as cauda equina syndrome, spondylolisthesis, segmental instability and large calcified disc herniation. However, our study was cross sectional questionnaire study which included non-surgical and previous surgical cases thus we did not explain in details for indication or contraindication of microscopic or endoscopic spine surgery.

**Comment 2: I think the author could include patients who underwent surgery for one year or more years in future studies. It can help to understand how the long-term outcomes of the two surgical methods influence patients' preferences.**

**Reply:** Thank you very much for your comment. We agreed and added this in our limitation in Discussion. Further study for long term outcomes of the two surgical methods are very interesting.

**Comment 3: The conclusion is too long, which should be shortened.**

**Reply:** We revised our conclusion which made it more concise as yours suggestion.

………………………………………………………………………………………………………….

**Reviewer #2:**

**Comment1:**

**Line 39: most patients prefer ELD (patient preference]**

**Line 62: ELD had same outcome with less complications than MLD [scientific preference].**

**Therefore, you should add in the conclusion that ELD is preferred scientifically and by patient’s preference and no need to ask for patient’s opinion in future, as ELD is preferred whenever it is available and possible.**

**Comment2:**

**Line 22, 65: There is no clear evidence for how to select the procedures. Why? (You mention later in 61- 62, line 208, 221 [Although the treatment outcomes and complications were not significantly different, ELD can reduce tissue injuries and reduce hospitalization].**

**Reply:**	Both ELD and MLD are minimal invasive spine surgery. From the literatures, the primary outcomes such as pain, function score, reoperation and complication are not statistically significant difference. The ELD may be more advantage in term of length of stay and blood loss as mention in Discussion. However, the different between them is not enough to affect the outcomes of the treatment such as the different in blood loss is about 45 cc [Reutten S et al Spine (Phila Pa 1976) 2008;33:931-9.] and no need to transfusion. Then we could not clearly advice the patients that which operation is better. That why we asked the patients opinion, especially, the patients who had experience of surgery compared with patients who had no experience of surgery and received information only from the documents.

**Comment 3:** **Line 138: what is the value of including the marital status, residency and academic degree in the questionnaire of this study?**

**Reply:** We hypothesize that some demographic data would affect the patient’s decision such as socioeconomic, family status or educational level. There are many information and methods of spine surgery can be found online which could affect the preference of patients. However, our results showed that the demographic data did not statistically influence the patient’s selection.

**Comment 4:** **Where are the patient information sheath? Like what is differences about wound size, time of surgery … between the two procedures in the information sheath. These information of course affect patient decision.**

**Reply:** The comparison information sheet between MLD and ELD was in Appendix1.

**Comment 5:** **Page 29, 30, 31: links: readers should read all informations of the subject in one file, not to follow a links. Please try to merge informations of the appendicular files with the main manuscript. It is difficult to publish this form of paper.**

**Reply:** There are many details in the information sheet and the questionnaire which could make the main manuscript too long. We would like to create links that can access the appendix 1 and the appendix 2 when click on the words.
